# Using Metaphors to Make Research Findings Meaningful

**DOI:** 10.1177/08445621221085555

**Published:** 2022-03-03

**Authors:** Rose Steele, Jennifer Baird, Betty Davies

**Affiliations:** 1 School of Nursing, Faculty of Health, York University, Toronto, Ontario, Canada; 2 Clinical Services Education and Research, Children’s Hospital Los Angeles, Los Angeles, CA, USA; 3 School of Nursing, 8205 University of Victoria, Victoria, British Columbia, Canada; 4 Department of Family Health Care Nursing at the University of California, San Francisco, USA

Researchers, educators, and funding agencies frequently lament that research is seldom read or put into practice by clinicians. Clinicians, on the other hand, note that they are busy and do not have the time or even the knowledge to read research articles that may be dense and filled with jargon. Traditional ways of disseminating knowledge are often insufficient; therefore, many funding agencies encourage researchers to find innovative ways to help their funded research be applied in practice. The Canadian Institutes of Health Research (CIHR; CIHR, 2012), for example, emphasized that varied strategies for knowledge translation, including non-academic modes of communication, are often needed to reach potential knowledge-user audiences beyond the research community. The CIHR encourage researchers to adapt their language of publication to fit target audiences and to present findings in alternative formats. In this column, we propose the use of metaphor as one innovative way to make knowledge useful for application in healthcare settings, so that, as noted by Straus et al. (2011), findings can be easily understood and capture the attention of the intended users of the knowledge.

As researchers with many years of combined experience and multiple funded studies, we were confident in our abilities to successfully complete a recent study in which we aimed to develop an empirically-grounded and theoretical conceptualization of what makes it possible for some healthcare providers, more than others, to engage in excellent interactions with parents of children with serious illness despite having similar time and other constraints. However, during our concurrent data analysis we realized that the dynamic complexity of what we were finding could not easily be expressed through our usual approaches. Therefore, we sought creative ways to make meaningful sense of the findings so that students, clinicians, educators, and administrators could understand and then use them. As we searched for the most suitable approach, we began to learn more about metaphors and eventually we chose a prairie windmill metaphor to make sense of the findings and bundle them together in the fullness of details (Davies et al., 2022). Our metaphor made the findings clearer and more manageable while also allowing various audiences to make sense of their own experiences of interactions with parents, patients, families, colleagues, and others:

The metaphor shows the whole of interaction, the movement back and forth of the many facets that are important. It captures the mystery of interaction, of the connection that really makes things happen. It's fun to play with because it really makes you think in a different way about excellence in interaction. ([study participant]; Davies et al., 2022, p. 13)

For many centuries, metaphors have frequently been used to express understanding of complex concepts. For example, as humans we know that when we talk about building bridges between people we are talking about the connections and not actual physical structures. Metaphors are useful for inviting people into worlds that they might not otherwise have seen. They can stimulate imagination, incite feelings, help people to see new meanings, and even lead to change. In qualitative research, metaphors can help simplify complex and/or multidimensional concepts through connecting one familiar concept to another familiar one, resulting in the comparison between the two concepts opening up new possibilities and perspectives (Schmitt, 2005). Metaphors provide structure to data and aid understanding of a familiar process in a new light. Thus, finding the right metaphor can help researchers describe complex findings in ways that others find meaningful.

We knew we had found the right metaphor when clinicians from many settings and disciplines, as well as parents, patients, and other family members, told us that the metaphor spoke to them and that the model made sense:

The windmill is really insightful, much more creative, dynamic, and transformational. I think what's brilliant to me about this model is that there are so many elements and to try to figure out which ones are connected at which level or layer and how they all work, the wholeness of it—I think it's really wonderful. ([study participant]; Davies et al., 2022, pp. 24–25)

As indicated by the previous quote, metaphors can be transformational and so can effect change. Effective change typically occurs incrementally, so if metaphors are used to tap the imagination and emotions of an audience, then a more evolutionary change may result that is, in the long run, more effective in putting new knowledge into practice. However, while the right metaphor can be extremely useful, it also is important to understand that using metaphors to translate research results is not just a new way of offering information. Rather, it is the comparison process within the use of metaphor that allows the audience to experience and understand one concept in terms of another; the metaphoric structure is what helps facts become interpretable or make sense (Richardson, 2003). We propose that if researchers are serious about helping practitioners and educators use research results, then they must present their findings in such a way that they touch or capture the personal experiences of practitioners and educators so they can find personal meaning in the new knowledge. Metaphors provide one intriguing approach to achieving this aim.
